# Experimental investigation of the multianticipation mechanism in commercial SAE level 2 automated driving vehicles and associated safety impact

**DOI:** 10.1016/j.aap.2024.107784

**Published:** 2024-12

**Authors:** Riccardo Donà, Konstantinos Mattas, Sandor Vass, Biagio Ciuffo

**Affiliations:** European Commission Joint Research Centre (JRC), 21047 Ispra, VA, Italy

**Keywords:** Adaptive cruise control, Car-following, Cut-out, Fuzzy safety metrics, Multianticipation, String stability, Traffic dynamics

## Abstract

Extensive experimental analyses concerned with Adaptive Cruise Control (ACC) have clearly shown that such systems have failed to deliver the promise of safe and traffic-flow effective car-following. On the contrary, large reaction times and poor string stability performances characterize commercial ACCs. While a huge research line is investigating the introduction of communication among vehicles to overcome the mentioned limitation, market adoption of connectivity-enhanced vehicles is struggling. In this context, an alternative approach based on multiple vehicle anticipation using RADAR only has emerged. Multianticipation is definitely not a new concept within the transportation community. However, until now, it was mainly associated with human driving. In the present manuscript, we demonstrate instead how, at least, one vehicle manufacturer has implemented multianticipation on a commercial vehicle. Following an in-house carried out testing campaign, we give an experimental characterization of the functioning of such a system including the potential impact on the flow and safety using a state-of-the-art fuzzy-logic safety performance model. The first results demonstrate that the vehicle under test reacted to one additional vehicle in front of the leader vehicle. Moreover, the actual realization appears to mainly target safety applications whereas there is only a marginal benefit on the string stability characteristics of the system. While we recorded a marginal string stability improvement (about 10 %), the minimum TTC was twice as large when multianticipation occurred with respect to the cases when that was not activated. Relevant Fuzzy Surrogate Safety Metrics further supported the safety argument.

## Highlights

• The work unveils the existence of multi-leader anticipation in commercial vehicles.
• Simulations and tests are performed to assess the multi-anticipation safety benefits.
• The specific multi-anticipation proves effective in reducing the risk of collision.
• Minor impacts on traffic flow as the mechanism is used as an active safety system.

## 1 Introduction

Automated driving technologies are making their way into the market. Adaptive Cruise Control (ACC) systems, which for many years have been available only for some high-end vehicles, are now offered for a wide range of different vehicles. ACC is only on SAE Level 1 of automation (SAE On-Road Automated Vehicle Standards Committee, 2021), or Level 2 when combined with lane-keeping automated features. Nevertheless, ACC has been expected to bring significant benefits to the highway traffic conditions, provided that the market penetration rate is high enough. Only recently, however, several research teams were able to perform experiments with commercial ACC systems and the consensus is that ACC is failing on the given promises for traffic flow and safety on motorways (Ciuffo, 2021, Shi and Li, 2021, Gunter, et al., 2020). Moreover, the latest NHTSA report shows worrisome outcomes regarding Advanced Driver Assistance Systems (ADAS) safety (NHTSA, 2022).

The failed expectations have been a motivation for further research in alternative solutions, and the main one is the use of connectivity (Hung and Zhang, 2022, Li et al., 2017). Indeed, there have been several research works on how connectivity can be used to benefit traffic safety and flow. Nevertheless, those failed expectations are providing space for important lessons to be learned. In the current state, traffic flow and traffic safety research are often carried out without the cooperation of the manufacturers. Therefore, assumptions regarding the functioning of ADAS systems must be made. In the case of ACC, it has been assumed that those systems would have negligible response time and they would be string stable (Kesting et al., 2008). Although those beliefs were reasonable at the time, when ACC systems became more widely available, experimental campaigns showed that their response time can be comparable to that of a human driver (Makridis et al., 2018), and for a wide range of their Operational Design Domain (ODD), they are string unstable (Gunter, et al., 2020, Makridis et al., 2021). This highlights the need to take the promises of future technological solutions with a grain of salt when the relevant expectations are not backed by real-world testing. Therefore, additional efforts in the experimental validation of all such assumptions are of paramount importance.

In this light, while connectivity still is the most promising and valuable solution, it has still not made its way to the market. Conversely, the time of its introduction is still unclear. Potential issues that can delay the mass deployment of connected-cooperative automated driving solutions have to do with cyber-safety (Khattak et al., 2021), the limited bandwidth available (Lyu, 2021), and the need for standardization, as right now different manufacturers may develop very different systems that can be incompatible for cooperation. On the other hand, an alternative solution, known as “multianticipation”, may not hold the same value of promises as connectivity, but its first applications are already on the market.

Multianticipation is the capability of adapting the control of the ego vehicle based on multiple leaders ahead during car-following maneuvers and has a high potential to increase capacity and traffic safety (Donà et al., xxxx). There is strong evidence that human drivers use multianticipation whenever visibility conditions allow (Lenz et al., 1999, Ossen and Hoogendoorn, 2006). Multianticipation has shown to have a beneficial effect both for stabilizing the traffic flow and, most importantly, on traffic safety (Sun et al., 2018, Ngoduy, 2015) and could potentially improve also ACC behavior (Donà et al., xxxx). However, for most of the commercially available ACC systems, there is no evidence to suggest that multianticipation is used, and although those details are proprietary, simulation models of the car-following behavior of commercial ACC systems, based on data, are not using information from more than one vehicle downstream (He et al., 2022, Milanés and Shladover, 2014, Gunter et al., 2019). Therefore, for this work, we assume that traditional ACC systems do not use multianticipation. That was the accepted reality until when Tesla came out with a release of its Autopilot system (v8.0) which is claimed (Tesla, xxxx) to react to the behaviors of more than one vehicle ahead taking advantage of the onboard perception system only.

**Fig. 1:**
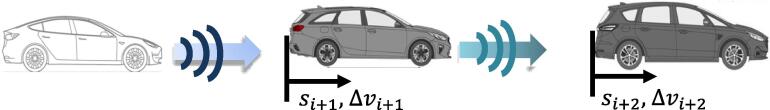
Traditional car-following with one-vehicle lookahead.

To put the mechanism into a graphical perspective, in a traditional ACC-based platoon every vehicle has only access to its immediate leader’s relative spacing $s_{i+1}$ and relative velocity ${\Delta v}_{i+1}$ as shown in Fig. 1.

**Fig. 2:**
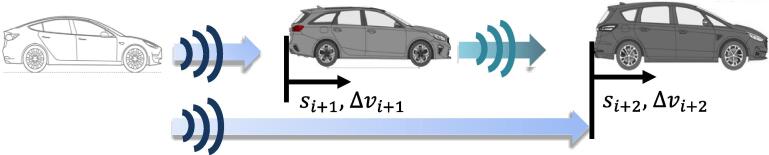
Multiple leader anticipation platoon using RADAR.

Introducing the multianticipation mechanism provides that the follower can now rely on its second leader bumper-to-bumper distance $s_{i+2}$ and relative velocity ${\Delta v}_{i+2}$ as in Fig. 2 while planning the driving policy.

In contrast to the Cooperative ACC (CACC) which uses connectivity, in a multianticipation scheme (hereafter referred to as “MACC”) the second leader’s information $s_{i+2},\Delta v_{i+2}$ is not gathered via broadcasting the vehicle state but via the RADAR’s waves multipath propagation effect (Holder, et al., 2018) which permits detecting obstacles that are not in the direct line-of-sight. In principle, this system would allow handling dangerous car-following scenarios when the in-between vehicle is tailgating its leader in a securer way, or when a traffic shockwave is approaching. In a similar way, cut-out scenarios could be handled more safely.

In this light, the present paper is describing our recent experimental campaign using the Tesla Model 3 MY 2021 model sold in the European market that has been stated to use multianticipation, hereafter referred to as the Vehicle Under Test (VUT). The scope of the paper is twofold. First, the multianticipation features are explored through experiments, to support the claim that MACC is possible for commercial vehicles and to provide insights into the observed behavior. With this, we hope to bring valuable information to researchers and modelers investigating such features, validate their assumptions, or produce evidence that some assumptions may not be suitable, and attract additional attention to MACC solutions, that seem more readily attainable than alternatives such as connectivity, for the time being. Secondly, the authors present an impact assessment on the traffic flow and safety. Regarding safety, the main focus is on rear-end collisions, which are the main type of accident that can be averted by the addition of multianticipation. The experimental campaign is divided into low-speed experiments inside the Joint Research Centre (JRC) campus in Ispra and high-speed on public highways around the area. While more extended experiments are required to reach a robust understanding of the impacts, especially regarding safety, this first preliminary work can bring some evidence to the intent of the developers of the specific solution, and the reasonable expectations of such a system. Eventually, we should point out that the ultimate goal of the work is not to provide judgment or to score the VUT-specific implementation of MACC which still remains a proprietary commercial technology.

The present paper is organized as follows. In section 2, we analyze the scientific literature concerning the use of multiple leaders’ anticipation. In section 3 we present the methodology we adopted to investigate VUT’s multianticipation implementation. We discuss the result in section 4 before drawing general considerations in section 5 together with policy recommendations. The conclusions are provided in section 6.

## 2 Literature overview

Multianticipation is a widely studied topic within the transportation research community, from Bexelius’ pivotal work during the late ‘60s (Bexelius, 1968) to very recent simulation-based contributions (Donà et al., 2022, Donà et al., 2023). Overall, two main lines of research have emerged throughout more than fifty years of scientific debate. The first line is concerned with the study of humans’ car-following behavior through microscopic simulation models involving multianticipation (Lenz et al., 1999, Ossen and Hoogendoorn, 2006, Farhi et al., 2012). The main outcome of such a research line was the appraisal of the stabilization effect on the traffic flow, as discussed by Monteil et al. (2014), yielded by multianticipation even under large reaction times (Treiber et al., 2007). In particular, Lenz et al. (1999) studied the stability properties of the Optimal Velocity Model (OVM, Bando et al., 1995) when up to three leaders are accounted for in the car following policy. Ossen and Hoogendoorn (2006) adopted an empirical-based approach to evaluate the degree of multianticipation which was found to be driver specific. Eventually, Farhi et al. (2012) identified the parameters of a piecewise linear anticipative model for the NGSIM (FHWA, 2022) database.

A parallel line of research was established among scientists studying inter-vehicle connectivity solutions (V2V), such as the CACC. The possibility of sharing vehicles’ information allows for several car-following schemes which are extensively discussed in, for instance, (Wang et al., 2018, Dey, 2016). The highlighted benefits of CACC with respect to ACC can be summarized as a higher capacity and better string stability properties which directly contribute to the safety of highway driving (Li et al., 2017). Moreover, inter-vehicles communication can also be exploited to signalize upcoming driving challenges, increase merging throughout, and handle traffic junctions (Elliott et al., 2019). Nonetheless, the market adoption of CACC solutions is virtually inexistent and overwhelmed by several challenges such as cybersecurity threats and missing supporting infrastructure (van der Heijden et al., 2017). Connectivity-based approaches have also been compared to a candidate formulation for multianticipative ACC in Donà et al. (2023) where it was shown how, under a penetration ratio below 40–50 % the multianticipative ACC can even outperform CACC unless platoon management techniques are enforced by the infrastructure operator.

As such, large-scale experimental campaigns involving CACC vehicles can only rely on prototypes (Shladover et al., 2012, Ploeg et al., 2011). That is in contrast with ACC practice, given the widespread adoption of the ACC technology which now allows for extensive investigations. Some central scientific contributions can be found Gunter, et al. (2020) and Makridis et al. (2021). In particular, Makridis et al. (2021) put a strong emphasis on the safety argument by emphasizing how the slow response of many ACC systems might yield to an unsafe situation in longer platoons.

Regarding the safety-relevant impacts of those solutions, the main focus is on rear-end collisions. While SAE Level 2 systems can also impact other accident types as they should include lane support systems (Cafiso and Pappalardo, 2020), rear-end collisions are the most frequent type of accident on freeways (Guo et al., 2019). Moreover, even simpler systems such as Forward Collision Warning (FCW) are shown to decrease rear-end collisions (Wang et al., 2016) even to 50 % if FCW is combined with Automatic Emergency Braking (AEB) (Li et al., 2017), according to police-reported crash data. Driving simulation data show an increase in the safety level, especially in the presence of fog (Wu et al., 2018). Overall, simulation-based experiments support that properly calibrated ACC systems can bring significant benefits regarding those accidents on freeways (Li et al., 2017, Rahman and Abdel-Aty, 2018), and even better results could be attainable using CACC, even if there is only one leading vehicle being tracked (Li et al., 2017, Rahman et al., 2019). A meta-analysis has shown that connectivity and automation have the potential to halve the total number of accidents in some countries (Wang et al., 2020). However, current technologies are not expected to decrease crash avoidance by more than 70 % (Yue et al., 2018). Studies, of the effects of MACC on rear-end collision, are missing, especially regarding real-world data, which have not been attainable so far, as MACC has just been introduced in commercial vehicles.

Considering the actual safety assessment of automated driving technology, several approaches have been presented in the literature. A recent RAND report effectively summarizes them in Blumenthal et al. (2020) by suggesting three classes:

• safety as a measurement;
• safety as a process;
• safety as a threshold.

In the current work we, take advantage of the first class, which is concerned with using either *lagging* measures or *leading* measures to assess the safety level of an Automated Driving System (ADS) technology. More in detail, lagging measures illustrate the actual count of critical events after they have occurred, whereas, leading measures are represented by metrics that are capable of accounting for pre-crash measures and/or the driving behavior of an ADS such as Surrogate Safety Metrics (SSMs).

Concerning rear-end, the most widely adopted SSM in both real-world and virtual experiments is the Time-To-Collision (TTC) (Hayward, 1972), which expressed how much time would elapse before a collision occurs given the instantaneous speed difference and distance. Based on the TTC, aggregate metrics have been proposed such as the Time Exposed TTC (TET), which measures how long a vehicle persists in a dangerous TTC regime, and the Time Integrated TTC (TIT), which measures the integral of TTC falling below a certain threshold over time. An overarching approach, which is capable of accounting for supplementary traffic scenarios in addition to rear-end collisions only, was presented by Intel/Mobileye in Shalev-Shwartz et al. (2022) under the nomenclature of Responsibility-Sensitive Safety (RSS). Shortly after a similar model was also proposed by NVIDIA with their Safety Force Field (Nister et al., 2019).

An issue affecting the TTC and its derived metrics is the variable threshold adopted to distinguish between critical and not critical events which ranges in the interval 1 s–4 s (Li et al., 2017, Mahmud et al., 2019). In order to overcome the definition of a crisp threshold value, fuzzy-logic based safety metrics have been proposed in the scientific literature. In particular, the effectiveness of TTC for rear-end collisions has been studied in Mattas (2020) as a comparison with the therein proposed Fuzzy Surrogate Safety Metrics (FSSM) for a set of car-following maneuvers.

## 3 Methodology

This section illustrates the methodological approach pursued to investigate the functioning and effectiveness of multianticipation implementation in a commercially available vehicle.

### 3.1 Supporting vehicles and instrumentation

Three vehicles were exploited during the testing campaign:

• Tesla Model 3 MY2021 for the EU Market equipped with the standard Autopilot kit (VUT);
• KIA Ceed MY2020, equipped with the ADAS kit featuring ACC + LKS;
• Ford S-Max MY2020, equipped with the standard Cruise Control (CC);

Additionally, a static vehicle target was used in the cut-out tests for safety reasons.

Given the specific application, there was no need to include additional vehicles in the platoon’s formation since two leaders are sufficient to detect multianticipation. Moreover, a longer platoon would have introduced additional testing difficulties due to potential string instability effects and longer spacing needed for experiments.

All the vehicles were instrumented using U-Blox GNSS units running at 10 Hz to record speeds and positions. The collected raw GNSS signals were post-processed offline and low-pass filtered to remove high frequency noise. The samples collected in the proximity of tunnels were removed to prevent outliers from affecting the quantitative analysis. Additionally, we used third-order spline fitting to get continuous jerk interpolation out of the speed profile.

### 3.2 Car-following tests

**Fig. 3:**
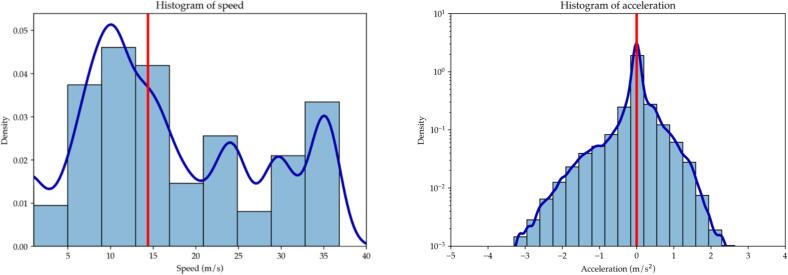
Histogram of speed (left) and acceleration (right).

The testing campaign started with a set of car-following (CF) experiments involving a 3-vehicle long platoon as in Fig. 1. The vehicles were driven by a team of JRC expert drivers involved in the market surveillance of motor vehicles that the JRC carries out on behalf of the European Commission. The drivers received clear instructions before starting the campaign and were made fully aware of the potential risks involved. They were also requested to always give priority to road safety and to proactively abort any experiment in case of possible risks for themselves and the surrounding road users. The authors were also in the three vehicles together with additional JRC technicians in order to be ready to adapt the test plans to the road and traffic conditions. The team performed multiple highway/motorway runs for a total of 138 km at a 61.59 km/h average speed (51.76 km/h median speed) to cover the 50–130 km/h speed range. The distribution of speeds and acceleration is reported in Fig. 3 where the three modal velocities corresponding to urban, interurban, and motorway driving can be denoted. The acceleration plot on the right denotes a median zero acceleration where the first and third quartiles are equal to −0.06 m/s^2^ and 0.08 respectively suggesting smooth driving scenarios.

**Fig. 4:**
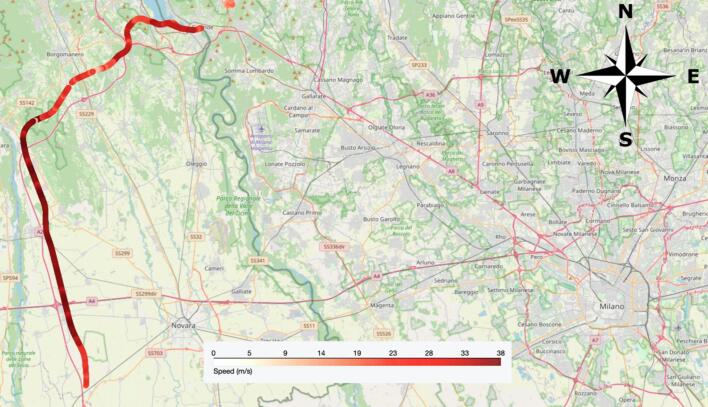
Map of test location.

The selected highway (A26 Vergiate-Vicolungo, IT) had three lanes per direction with physical separation among opposite traveling lanes. The tests were performed during the day on both directions to compensate for possible effects of slope and wind. The map of the recorded trajectories is shown in Fig. 4. The platoon of vehicles stayed on the rightmost lane to minimize interference with other vehicles and the tests were executed during off-peak hours. The drivers had received precise instructions concerning the execution of the tests and were assisted by co-pilots in constant radio communication among each other. 31 relevant events have been recorded in total.

The testing procedure was such that the last follower (the VUT) was using the Autopilot throughout the whole experimental campaign, the vehicle in the middle alternated between ACC (type “A” tests) and cruise control (CC, type “B” tests), whereas the leader traveled in CC mode expect when the perturbations were manually induced. Before any perturbation occurred, the platoon was firstly stabilized to make sure steady car-follow behavior is obtained. Then, the leader induced a velocity perturbation of 20 km/h. The in-between vehicle switched between ACC and CC depending on the type of experiment pursued. In the ACC mode the perturbation is propagated to the last vehicle whereas, in CC mode, the middle vehicle approaches the leader until dangerous tailgating conditions are reached before the driver’s braking intervention.

The first kind of experiment (middle vehicle in ACC mode, “CF-A”) is mainly aimed at assessing the string stability properties of the vehicles making up the platoon. Conversely, the second variation (“CF-B”) is directly targeting identifying any multianticipation capability of the VUT’s Autopilot. For all the second variation experiments, the VUT’s Autonomous Emergency Braking System (AEBS) system was set to “Anticipation mode”, whereas, for the first variation, we alternated between “Anticipation” and normal operation to explore if multianticipation also affects traffic flow.

### 3.3 Cut-outs tests

The second class of experiments is based on the cut-out (CO) scenario. In a cut-out scenario, the ego-vehicle is following in steady-state condition a leader vehicle when, suddenly, the leader vehicle performs a lane-change maneuver to avoid an obstacle in the current lane. Due to the higher criticality, cut-out tests were performed in JRC research facility in Ispra (VA), IT leveraging a static soft target to minimize harm in case of collision. The driver has received proper training a was assisted by supporting signals that indicated the position of the static obstacle even when not directly visible. A total of 12 valid scenarios were recorded.

**Fig. 5:**
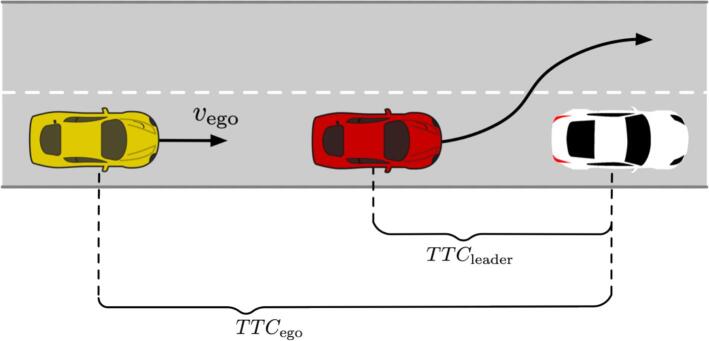
Cut-out scenario.

A schematic visualization of such a set-up is displayed in Fig. 5, where the ego-vehicle is represented by the yellow car, the leader vehicle is the red car whereas the white car is the static obstacle. In our realization of the experiments, we used the Tesla featuring the AEBS in anticipation mode as the ego-vehicle, the Ford S-Max as a leader, and the static target as an obstacle.

The investigations based on the cut-out scenarios were aimed at detecting whether multianticipation could be beneficial in addressing safely the mentioned scenario when the leader vehicle carries out the lane-change in the proximity of the target. Given the limited space available in the JRC facility, cut-out experiments proved to be limited to a maximum speed of 10 m/s.

### 3.4 Car-following simulation analysis

To enrich the scope of the analysis, we carried out a simulation study where an artifact representing the reversed engineered Tesla *partial* MACC behavior is compared to a traditional ACC and to a theoretical *full* MACC. The virtual tests are based on stochastic car-following virtual experiments where a leader vehicle introduces a perturbation starting from steady-state conditions. The leader-induced perturbations are derived from the “highD” dataset (Krajewski et al., 2018), a drone-collected set of trajectories on German highways thus realistic and representative of ACCs target design domain. The virtual platoon’s length has been fixed to 10 vehicles. Initially, all of those are considered ACC-operated and, subsequently, MACC-equipped vehicles, either fully or partial, are stochastically introduced within the platoon to monitor the effect on traffic flow and safety.

We simulate the baseline ACC car-following behavior using the well-known linear controller model under the constant time headway (CTH) policy:

(1) $$u_{\mathrm{ACC}}\left(t\right)=k_{d}\left(v_{L}\left(t-T\right)-v\left(t\right)\right)+k_{p}\left(s\left(t-T\right)-t_{g}v\left(t\right)-\eta \right)$$

where $u_{\mathrm{ACC}}$ is the predicted acceleration, $k_{d}$ and $k_{p}$ are the controller’s gains, T the perception delay, η the standstill distance, $v_{L}$ the leader’s speed, v the ego vehicle speed, s the bumper-to-bumper distance, and $t_{g}$ the desired time-gap.

With regards to the multianticipative mathematical formulation, the model presented in Donà et al. (2022) is here adopted. In comparison to the ACC linear controller, the full MACC model has additional terms in the control law, highlighted in bold, to include the role of the second leader:

(2) $$u_{\mathrm{MACC}-full}\left(t\right)=k_{d}\left(v_{L1}\left(t-T\right)-v\left(t\right)\right)+k_{p}\left(s_{1}\left(t-T\right)-t_{g}v\left(t\right)-\eta )\right)+w_{L2}\left(k_{d}\left(v_{L2}\left(t-T\right)-v\left(t\right)\right)+k_{p}\left(s_{2}\left(t-T\right)-2t_{g}v\left(t\right)-\eta \right)\right).$$

The $w_{L2}$ parameter modulates the relative importance of the second leader and was assumed as constant and equal to 1.0 thus assigning to the second leader equal importance as of the first one. $v_{L2}$ and $s_{2}$ are the second leader speed and spacing, respectively.

The partially anticipative MACC, which resembles the Tesla behavior, is given by a similar mathematical formulation as the full MACC where, however, an activation ϕ function and a saturation mechanism are introduced to replicate the behavior observed for the particular vehicle:

(3) $$u_{\mathrm{MACC}-partial}\left(t\right)=k_{d}\left(v_{L1}\left(t-T\right)-v\left(t\right)\right)+k_{p}\left(s_{1}\left(t-T\right)-t_{g}v\left(t\right)-\eta )\right)+\phi \left(s_{2},v,v_{L2}\right)\max\left(w_{L2}\left(k_{d}\left(v_{L2}\left(t-T\right)-v\left(t\right)\right)+k_{p}\left(s_{2}\left(t-T\right)-2t_{g}v\left(t\right)-\eta \right)\right),-2\right).$$

The activation function ϕ triggers the multianticipation behavior when certain conditions are valid whereas the saturation function makes sure that the individual contribution coming from the multianticipative term does not exceed a deceleration effort of 2 m/s^2^ as observed in the empirical data. The triggering function relies on the computation of the TTC with respect to the second leader as of:

(4) $$\phi \left(s_{2},v,v_{L2}\right)=\left\{\begin{array}{c} 1if\frac{s_{2}}{v-v_{L2}}<10\\ 0if\frac{s_{2}}{v-v_{L2}}\geq 10 \end{array}.\right)$$

In particular, the MACC is enabled when the TTC is lower than 10 s as it was experienced during the tests. In all the other cases, the “MACC-partial” will behave exactly like the traditional “ACC”.

The calibrated parameters for the car-following model are reported in Table 1. The simulation framework leverages a stochastic approach where each parameter is sampled from the ranges in the Table by assuming uniform distributions. The stochastic approach aims to make sure that the assessment is robust and not only valid for a given selection of parameters.

**Table 1:** Simulation study calibrated parameters.

Model	$k_{p}$	$k_{d}$	W_L2_ (−)	t_g_ (S)	T (s)	η (m)
ACC	[0.03, 0.1]	[0.25, 0.70]	−	[1.20, 2.00]	[0.7, 1.5]	[2.0, 3.0]
MACC-partial	[0.03, 0.1]	[0.25, 0.70]	1.0	[1.20, 2.00]	[0.7, 1.5]	[2.0, 3.0]
MACC-full	[0.03, 0.1]	[0.25, 0.70]	1.0	[1.20, 2.00]	[0.7, 1.5]	[2.0, 3.0]

The penetration rate of the MACC takes four discrete values: 0 %, 20 %, 50 %, and 100 %; a set of 15 perturbations from the highD dataset are considered each one repeated 30 times by drawing random parameters from the ranges in Table 1. As such, a total number of 3,600 simulations are performed corresponding to 4 penetration rates multiplied by 15 perturbations times 30 repetitions times the 2 MACC technologies postulated.

### 3.5 Surrogate safety metrics

In order to quantify any potential safety gain by using multianticipation, we exploited state-of-the-art safety metrics to assess the criticality of the rear-end potential collision events.

The first is the widely established TTC, which is computed as the ratio between the bumper-to-bumper distance and the speed difference

(5) $$\mathrm{TTC}=\frac{s}{v_{F}-v_{L}}.$$

Deriving from the TTC, the TET (Minderhoud and Bovy, 2001) computes the exposure to critical TTC by accumulating the cases where risky situations were encountered based on a given TTC threshold:

(6) $$\mathrm{TET}=\sum_{i=1}^{N}TTC\left(i\right)<TTC_{\mathrm{threshold}}.$$

The TET metric has been adopted in the car-following simulation investigation for estimating the safety outcome. Since the simulations were all equally long it was then possible to compare the percentage of exposure by normalizing according to the total number of simulation steps. In our work, we assumed a $\mathrm{TT}C_{\mathrm{threshold}}=3.0$ s.

The additional metrics for the safety assessment are the Fuzzy Surrogate Safety Metrics (FSSM) (Mattas, 2020). The use of fuzzy logic is addressing the issue of crisp thresholds in most traditional surrogate safety metrics. Therefore, the classification results in conditions that are certainly unsafe (with a membership value of 1), certainly safe (unsafe with a membership value of 0), and conditions with a degree of unsafety (with a membership value from 0 to 1). Two different FSSM are defined, Proactive Fuzzy Safety (PFS) and Critical Fuzzy Safety (CFS). PFS is corresponding to a definition of safety closer to that of the Vienna Convention on Road Traffic, “a collision between vehicles can be avoided if the vehicle in front performs an emergency brake” (United Nations Economic and Social Council, 1968). Assuming a hard deceleration of the leader vehicle, PFS evaluates the ability of the ego-vehicle to react and avoid a collision. Therefore, PFS identifies cases of tailgating, when there is no imminent danger of an accident. On the other hand, CFS evaluates situations in which there is an imminent danger, and evasive actions are required. The exact formulation of PFS and CFS, from Mattas et al. (Mattas, 2020), is presented in the Annex.

### 3.6 Traffic metrics

A parallel assessment procedure is related to the traffic flow, as we want to quantify if any safety increase is obtained at the expense of a traffic flow decrease.

String stability refers to the ability of the platoon to dampen the magnitude of oncoming oscillations (Wilson and Ward, 2011). For heterogeneous platoons of vehicles, the definition of weak string stability has been widely used, looking at the change in the magnitude of the perturbation only on the first and the last vehicle of the platoon (Montanino et al., 2021). We investigated the effectiveness of the ACC systems via computing the “weak” string stability ($w_{\mathrm{ss}}$) metric after every perturbation. In particular, we considered the ratio between the follower’s velocity ($v_{F}$) disruption divided by the magnitude of the perturbation induced by the leader $(v_{L}$)

(7) $$w_{\mathrm{ss}}=\frac{v_{L,0}-v_{F,min,}}{v_{L,0}-v_{L,min,}}.$$

If a perturbation yields a $w_{\mathrm{ss}}>1$ it is considered string unstable since the velocity perturbation gets amplified upstream. Vice versa, $w_{\mathrm{ss}}<1$ implies a reduction of the perturbation’s magnitude thus string stable behavior is obtained.

Moreover, we computed the reaction time via iteratively time-shifting the acceleration profile of the leader and taking the argmax() of the resulting vector of Pearson correlation as in Makridis et al. (2018).

(8) $$\arg\max_{T}(pearson\left(\Delta v\left[t-T\right],{\mathrm{acc}}_{F}\left[t\right]\right)$$

### 3.7 Modeling assumptions for baseline establishment

The computation of the PFS and CFS demands establishing a reaction time. In our work, we assumed a reaction time equal to 0.5 s as a reference value as a trade-off solution between the comfortably-tuned ACCs, which have delays in the order of 1.0 s (Makridis et al., 2018), and the more responsive emergency braking systems. Moreover, we set the comfortable deceleration to 2.0 m/s^2^ (Shladover et al., 2012); whereas we assumed the maximum deceleration equal to 6.0 m/s^2^, despite the actual vehicles could manage stronger braking, to account for a conservative tire-road friction coefficient. The deceleration assumptions are validated by the distributions presented in the results section.

For all tests, differently from simulation experiments, the velocity signal obtained was never exactly stable, either because of very small fluctuations of the actual speed, or the noise of the sensing equipment. Hence, we assumed the starting time for the deceleration phase to begin when the recorded deceleration exceeded 0.5 m/s^2^, since smaller decelerations are possible also for steady-state conditions.

For the VUT, we could not obtain the exact time-step when it was able to get information for the vehicles downstream. The dashboard representation for the user would show the existence of two vehicles upstream for most of the experiments, but not always. Therefore, it can be assumed that, in some cases, the VUT was not able to identify or locate the leader upstream. However, even when it appeared, this graphical interface may not be the most reliable representation, as it can be the case that the position of the upstream leader could be temporarily extrapolated. Therefore, we chose not to collect or process the dashboard indications, as the authors do not have the insight to correctly interpret this information.

Moreover, for the CF-B experiments, we computed the PFS as a reference safety metric since there is not an imminent risk of collision for the VUT with respect to its first leader.

**Fig. 6:**
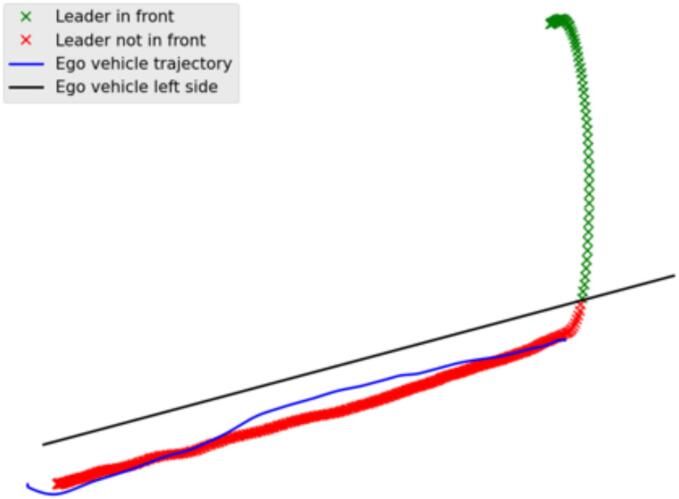
Methodology for establishing the leader in the cut-out scenario.

Concerning instead the cut-out tests, we assumed the leader vehicle to be in front of the VUT until when the center of mass of the leader lies within the projected lateral envelope of the VUT as depicted in Fig. 6. In addition, we computed the CFS as a relevant safety metric since criticality conditions occur given the presence of an occluded static obstacle.

We also enriched the discussion by envisaging how a non-multianticipative vehicle would have behaved in the considered scenarios via simulation. For the CF-B, we assumed that a non-multianticipative vehicle would have kept constant speed until its immediate leader braked as there would have been no other reason to slow down. We then calculated the PFS when the TTC between the first vehicle and the middle vehicle was the smallest (higher risk of collision) throughout the perturbation maneuver, until the middle vehicle brakes.

Conversely, in the COs, a non-multianticipative vehicle would keep the traveling velocity until its leader did not perform the actual cut-out maneuver and accumulates enough lateral displacement according to Fig. 6. In such scenarios, we calculated the CFS just before the leader leaves.

## 4 Results

### 4.1 Basic characteristics of multianticipation

**Fig. 7:**
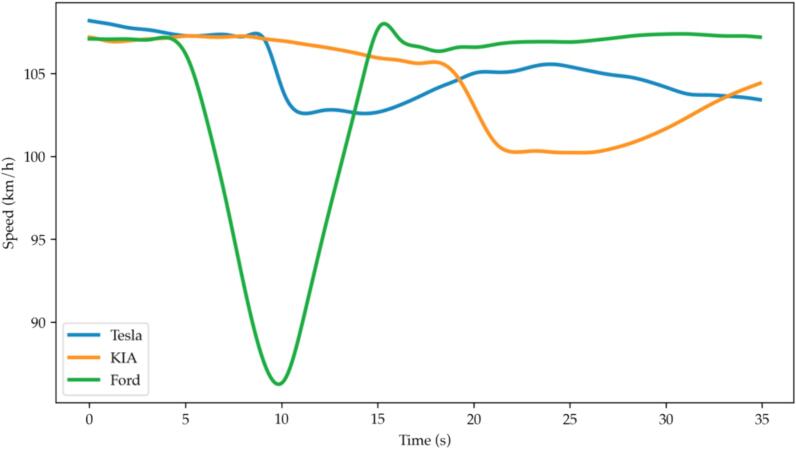
Multianticipation example from a car-follow experiment.

**Fig. 8:**
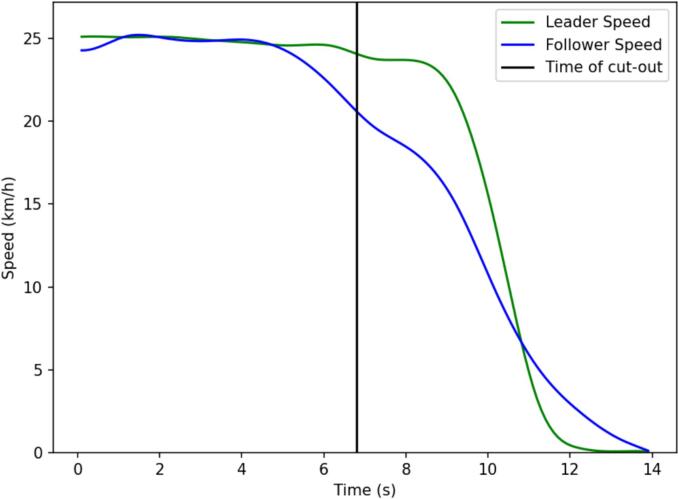
Multianticipation example from a cut-out variant scenario.

In this subsection, we present the evidence of multianticipation in the observed data, and we evaluate the dynamic characteristics of this behavior. In Fig. 7 and Fig. 8 two cases of multianticipative behavior are presented from the car-following and the cut-out scenarios. In Fig. 7, the leader vehicle is the Ford and its speed is denoted by the green line. The speed of the leader vehicle is almost constant until it creates a perturbation in the form of a deceleration, followed by an acceleration to the set speed. The second vehicle in the platoon is the KIA, whose speed is denoted by the orange line. The driver of the KIA is using the normal CC, to keep a constant speed for as long as there is no significant safety risk. The speed of the third vehicle, the VUT, is denoted by the green line. The deceleration of the VUT started well before any meaningful deceleration of its predecessor, and decelerated to a lower speed, to increase the distance to the predecessor. Afterward, when the KIA decelerated, the last vehicle had already created a separation that was enough, not to need any additional deceleration. Moreover, the VUT started the deceleration several seconds after the leading vehicle started the perturbation when the separation between the two leading vehicles was already small. This evidence suggests that the MACC algorithm may be implemented not to react directly to the speed of the leading vehicle, but to the spacing. Therefore, it seems it is designed with safety in mind, reacting to unsafe conditions, and not to facilitate the flow, as it would have to react also to the speed change.

In Fig. 8, a case of multianticipation is presented from the cut-out experiments. The leading vehicle leaves the trajectory at the time instance denoted by the black line. The speed of the VUT is denoted by the blue line, while the leader vehicle’s speed is denoted by the green line. The VUT, being the follower vehicle had already detected the static target vehicle, as it was indicated in the dashboard, and started decelerating before the leader vehicle even started the turning maneuver, to leave the trajectory. Again, this can be achieved only if the vehicle's control strategy considers more than one leading vehicle.

**Fig. 9:**
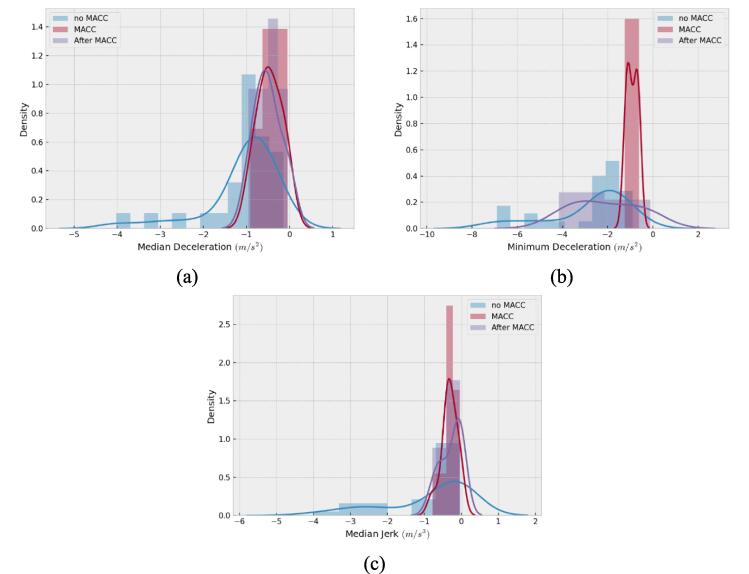
Distributions of (a) median deceleration, (b) minimum deceleration, (c) median jerk, for all the events.

Since, to the best of the authors' knowledge, this is the first documented evidence of multianticipation in commercially available automated driving systems in the literature, some useful characteristics are presented. The events have been classified according to the existence or not of multianticipation deceleration, regardless of the experiment type, car-following, or cut-out. The median deceleration, minimum deceleration, and median jerk are evaluated, for the cases where no multianticipation is observed, and for the multianticipation, they have been further divided into the time duration of the multianticipation, and the time duration after the multianticipation, when the leader has already decelerated or left the trajectory. The distributions are presented respectively in Fig. 9(a)–(c). The median deceleration is much smaller during and after the multianticipation part of the deceleration than the median deceleration for the cases where no multianticipation was observed. Thus, multianticipation can improve comfort, in real-world applications. The distributions of the minimum decelerations show similar results, as the minimum deceleration was frequently harder when no multianticipation was observed. On the other side, the minimum deceleration during the multianticipation was never below −2 m/s^2^, which could be a design constraint by the manufacturer, to avoid unexpected hard decelerations that could be uncomfortable for the passengers. Finally, the median jerk values are more comfortable when multianticipation is observed, with the tail end of the distribution of the no-multianticipation cases dropping to smaller values.

### 4.2 Car-following tests

For the more detailed investigations, we split the elucidation of the car-following results according to the two variants (CF-A vs*.* CF-B) explained in the methodology given the different considerations that apply to the two scenarios.

#### 4.2.1 CF-A traffic-related results

We computed the weak string stability metric for both the VUT, considering the KIA as its immediate leader, and the KIA, considering the Ford as a leader for all the 21 clean perturbations recorded. For the VUT, we keep a neat separation between cases when the “anticipative” mode (MACC) of the AEBS was used.

**Fig. 10:**
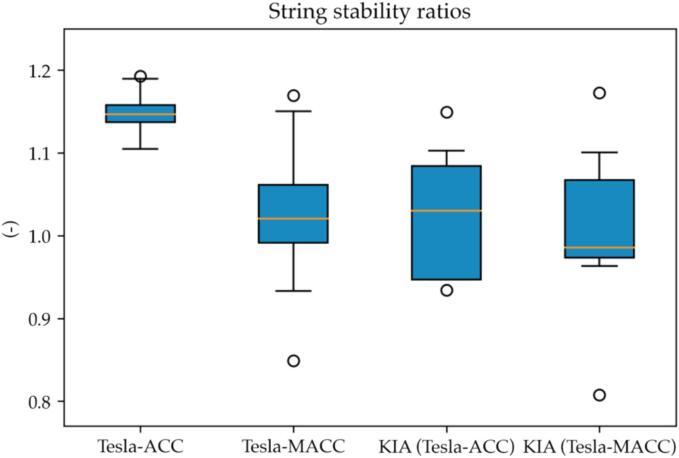
Tesla’s string stability distributions.

The interquartile ranges for the cases “ACC” and “MACC” are shown in the first two whiskers in Fig. 10. As it can be noticed, in both cases the median value is higher than 1, indicating the VUT is *not* string stable. Nonetheless, we experienced a reduction of the median string stability ratio while enabling the MACC mode, albeit the results are more sparse in such a setting.

Considering instead the KIA, one can grasp from Fig. 10 how the middle vehicle (KIA) does not show a significant difference in the behavior for the “ACC” and “MACC” settings of the VUT. This fact suggests that the different behavior of the VUT cannot be explained by the different behavior of its leader. On the contrary, the VUT managed to cope in a more stable way when used in MACC mode.

The visual clue about the distributions of $w_{\mathrm{ss}}$ being the same for the KIA and different for the VUT can be further motivated by computing the *t*-test for each pair of distributions. The result of the computation is reported in Table 2.

**Table 2:** T-test values for the weak string-stability distributions. MACC vs ACC.

Vehicle	p-value	t-statistic
VUT	0.1537	1.4860
KIA	0.6941	0.4016

As Table 2 details, the correlation level between the KIA’s weak string stability distributions is much higher than the VUT’s. Nonetheless, the *t*-test does not allow ruling out the null-hypothesis stating that the VUT’s distribution has the same mean.

Additional evidence suggesting the different functioning of the VUT’s ACC is represented by the median time-headway between the two types of experiments. For the ACC cases, a median of $t_{h}=$ 0.937 (s) was reported whereas for the MACC cases the median headway was 0.861 (s). Given the limited discrepancy in the headway, we can assume that the better string-stability figures reported for the MACC cannot be ascribed to a different headway setting while enabling the anticipation mode.

The reaction time can be computed using (8) for the list of recorded perturbations. We kept strict separation between the occurrences when MACC was enabled and traditional ACC operation, similarly to the previous paragraph.

**Fig. 11:**
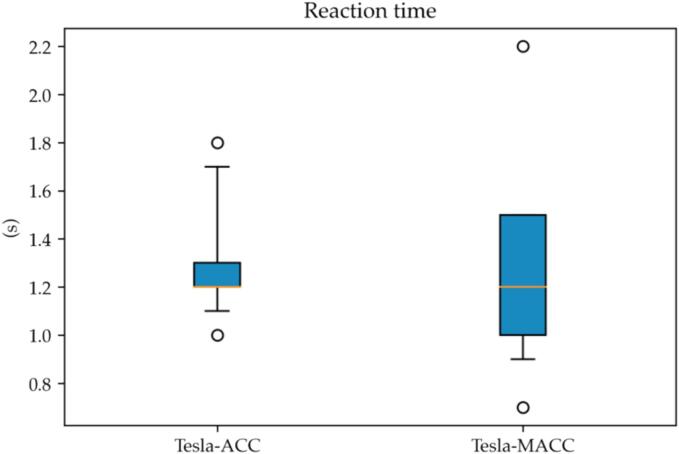
Reaction time interquartile plot. VUT MACC vs ACC.

From Fig. 11, it can be noticed how negligible differences exist in the reaction time for the two settings configurations. The computation of the *t*-test yielded, in fact, a p-value equal to 0.999. The (global) median computed value of the VUT’s reaction time turned out to be 1.2 s, a slightly lower value than averagely what averagely observed in (Makridis et al., 2018).

#### 4.2.2 CF-B safety-related results

Concerning instead the scenarios where the first leader applied a perturbation without a braking action by the in-between vehicle, we have recorded 10 events.

**Fig. 12:**
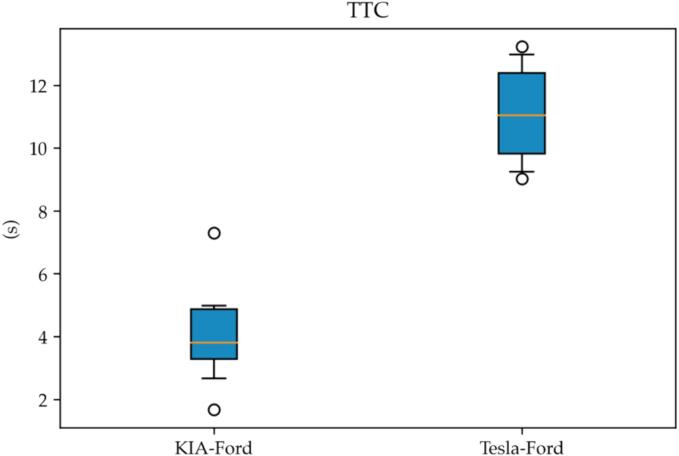
TTC for variant car-following tests.

The effectiveness of such behavior can be measured by computing the distribution of the TTCs for each pair of leader–follower as shown in Fig. 12.

As it can be visually grasped, the TTC for the VUT with respect to its second leader is consistently higher than 10.0 s. Moreover, we can claim that braking action is initiated by the second leader (Ford) as TTC is lower with respect to the first leader (KIA).

**Fig. 13:**
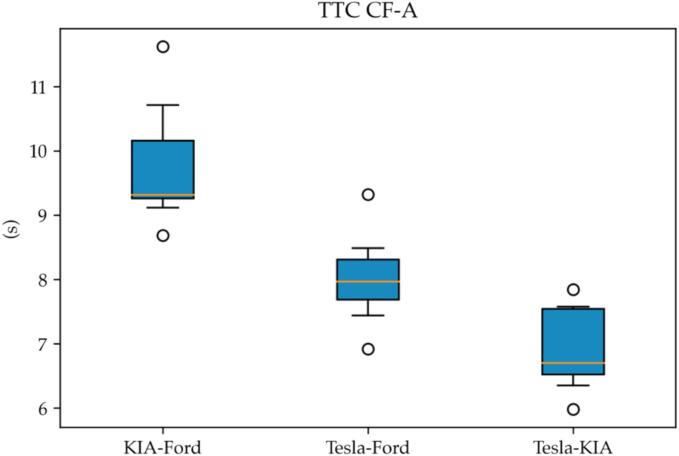
TTC distributions for ACC-based car-following.

The same consideration does not apply when studying the TTC for the traditional ACC-based car-following test (CF-A) where multianticipation is not enabled as shown in Fig. 13.

Interestingly, in such cases, the VUT uses a lower TTC with respect to both the leaders despite the actual trend of TTCs being coherent with the relative spacing of the vehicles, *i.e.*, higher for the second leader with respect to the first leader. Such a “target switching” behavior is a clear signal of a multianticipation feature. Moreover, it suggests that the implementation has been carried out with a safety goal in mind.

The safety argument is supplemented by the study of the PFS metric for the selection of CF-B scenarios. In particular, the study is conducted as a comparison between the recorded PFS and the extrapolated PFS which stems from the assumptions in Section 3. The extrapolated PFS is representative of how a vehicle with no multianticipation would have behaved in the same scenario. The maximum membership value of PFS is obtained. For the car-following events, we opted to evaluate the PFS, as the situation is never really critical. The results of the computations are presented in Table 3.

**Table 3:** Recorded and extrapolated PFSs for the CF-B scenarios.

Event ID	Duration (s)	Recorded PFS	Extrapolated PFS
1	1.9	0.934	0.965
2	2.5	0.925	1
3	−	0.892	−
4	−	0.915	−
5	−	0.928	−
6	10.1	0.925	0.977
7	−	0.959	−
8	−	0.930	−
9	2.9	0.972	1
10	1.4	0.925	0.980

The results show how the PFS is getting maximum membership values close to 1. This means that in case of a hard deceleration of the KIA, the VUT would have to decelerate with almost 6 m/s^2^ to avoid an accident. Therefore it would require a hard, but manageable deceleration. This is reasonable, considering that the minimum distance setting has been used. The PFS never gets the membership value 1. That would mean that 6 m/s^2^ of deceleration would not be enough. However, in two out of the five cases in which a multianticipative reaction was observed, the extrapolated PFS recorded a value of 1. In the remaining cases, the maximum extrapolated PFS was higher than what was observed for the cases in which no multianticipation deceleration is observed. Overall, the VUT managed to reduce the PFS by as much as 5 % on average.

### 4.3 Cut-outs experiments

**Fig. 14:**
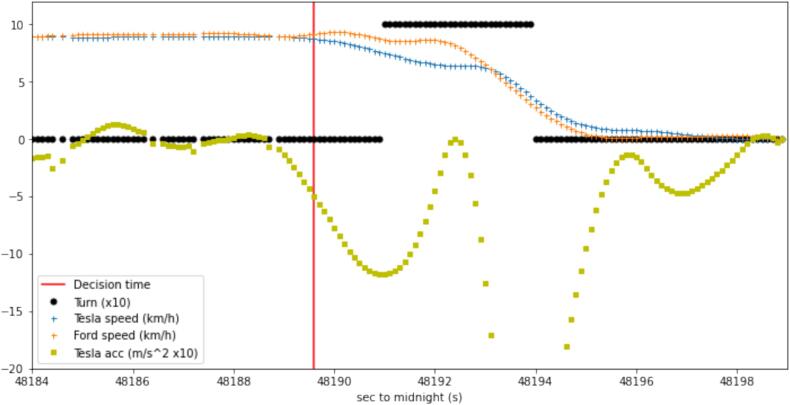
Cut-out multianticipation event.

The cut-out experiments investigate the effectiveness of multianticipation in addressing a challenging driving scenario with a limited deceleration effort. An example of the velocity profiles for the VUT and its cutting-out leader is shown in Fig. 14, together with the deceleration policy adopted by the VUT when the AEBS was set to use anticipative mode. The anticipative situation represented in Fig. 14 was not obtained throughout all the repetitions of the scenario. In fact, in the 12 experiments, only three cases showed clear multianticipation. On the contrary, in the remaining repetitions, we did not report any multianticipation and a strong deceleration was required to avoid colliding with the target.

The velocity profiles reported for the VUT were compared against a non-multianticipative baseline simulation model according to the assumptions highlighted in Section 3. The results are presented in Table 4 for the selection of scenarios which showed a significant multianticipative effect. For this case, the CFS metric is evaluated. The target is already static and the vehicle has to decelerate in order not to collide with the soft target. Therefore, the VUT has to take action to avoid an imminent collision risk, and the critical safety metric is more appropriate.

**Table 4:** Recorded and extrapolated CFSs for the CO scenarios.

Event ID	Velocity (km/h)	Recorded CFS	Extrapolated CFS
1	10	0	−
2	15	0	−
3	15	0	−
4	20	0	−
5	20	0	−
6	25	0	−
7	25	0	−
8	30	0	−
9	30	0	0.288
10	35	0.542	−
11	35	0	0.633
12	35	0	0.402

As of Table 4, the introduction of multianticipation allowed for a substantial reduction of the CFS. Moreover, when multianticipation occurs, CFS remains zero throughout all the experiments, so the VUT never has to decelerate harder than the comfortable deceleration value of 2 m/s^2^. For those cases, the extrapolated CFS shows that a harder deceleration would be needed if multianticipation did not kick in. On the other side, we reported one case when the CFS was higher than 0.5 with the multianticipation not being activated. Still, a collision is avoided, and the required deceleration is always less than 6 m/s^2^.

**Fig. 15:**
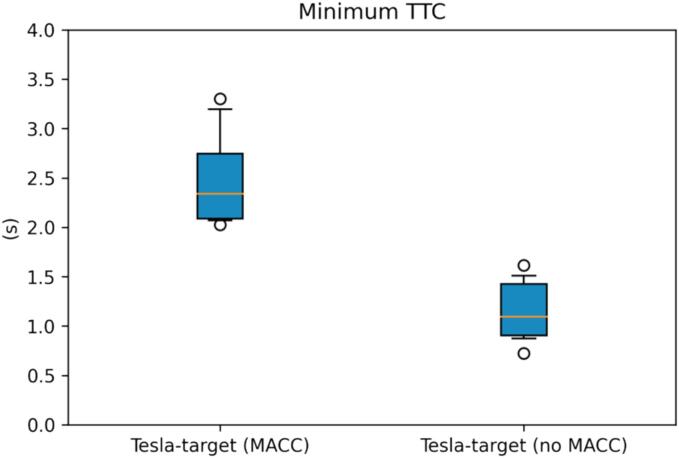
CO minimum TTC interquartile plot, MACC vs. no-MACC.

**Fig. 16:**
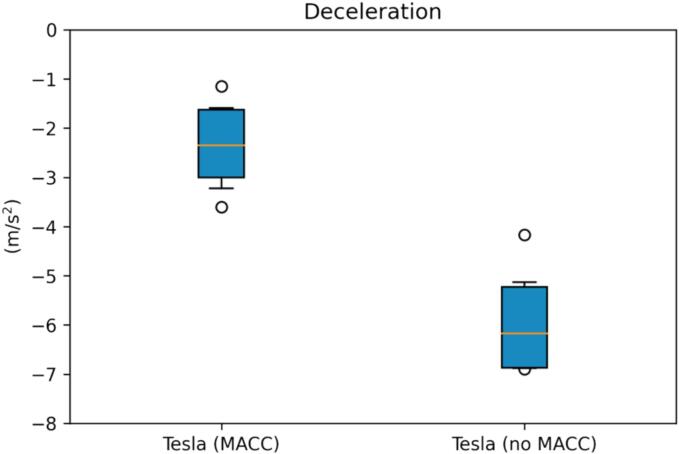
CO minimum acceleration interquartile plot, MACC vs. no-MACC.

The effectiveness of multianticipation can be additionally grasped from Fig. 15 and Fig. 16, where the interquartile charts for the minimum TTC and maximum deceleration effort are shown respectively. The median TTC when multianticipation took place is 2.13 times higher with respect to the non-multianticipative cases. Similarly, the minimum acceleration reduces, in absolute value, by about 60 % for the MACC events.

### 4.4 Car-following simulation results

**Fig. 17:**
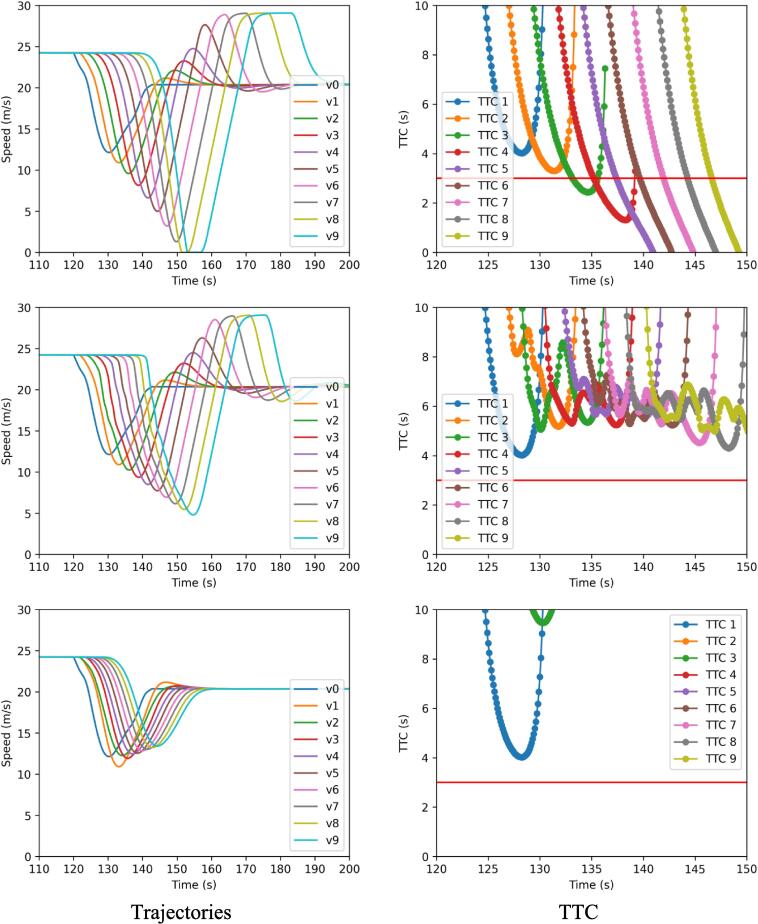
Trajectories and TTC for a reference perturbation: ACC (top), MACC-partial (middle), and MACC-full (bottom).

Fig. 17 provides a graphical visualization of the trajectories (left) and TTC (right) for a reference perturbation for the three car-following models considered: ACC (top), MACC-partial (middle, Tesla-inspired simulation artifact), and MACC-full (bottom) for a 100 % penetration rate in case of the MACCs. All the controllers have been calibrated using the same set of parameters to make sure that any difference observed can be ascribed to the different control laws only thus isolating the impact of multianticipation. A visual inspection of the data reveals how both the multianticipative solutions managed to reduce the magnitude of the perturbation being propagated downstream thus achieving better string stability with respect to the normal ACC. However, the full MACC proves much more effective in stabilizing the platoon. In the TTC charts, it is evident how the ACC fails to manage the scenario and 5 virtual collisions are reported at the tail of the platoon as the TTC reaches zero. On the contrary, even the partially multianticipative MACC can manage to keep the TTC above a critical value of 3 s. Eventually, the full MACC provides for an even larger TTC apart from the first follower which cannot use multianticipation due to having only one leader in front.

**Fig. 18:**
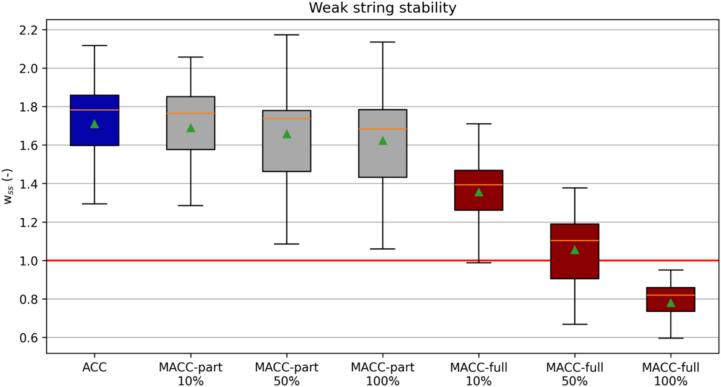
Aggregated simulation results.

**Fig. 19:**
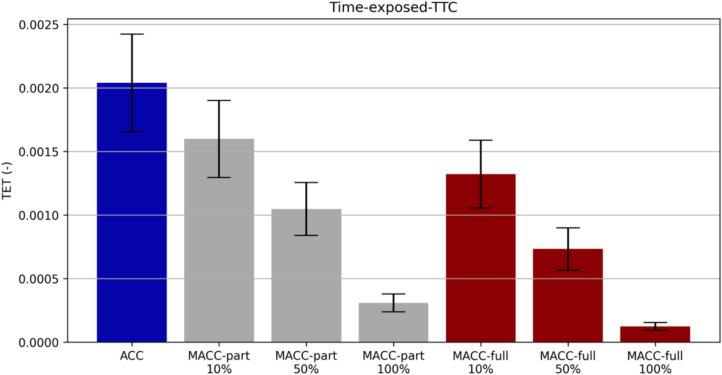
Time-exposed-TTC.

The considerations drawn in Fig. 17 for a particular perturbation find additional supporting evidence from the aggregated simulation outcome charts Fig. 18 and Fig. 19 where the results of the stochastic assessment are shown. More specifically, Fig. 18 illustrates the weak string stability boxplot chart whereas Fig. 19 summarizes the average TET together with the variance associated with. The weak string stability chart shows how the partial MACC (grey boxes) can only marginally contribute to increase the stability of the platoon with respect to the baseline ACC (blue box). On the contrary, the full-MACC is much more effective and the 100 % penetration case can achieve full string stable platooning for all the perturbations considered.

The situation changes when safety is considered as of the TET chart. In this case, the MACC-partial, albeit still less effective than the MACC-full, can substantially increase safety and the effectiveness is proportional to the rate of adoption.

### 4.5 Summary evaluation

A summary report of the results is given in Table 5. Whenever only partial data was available, only the change in performance was computed only for the corresponding cases where both KPIs were applicable. The fuzzy-based metrics PFS, CFS have been labeled as “*” since their value was estimated in the case of the ACC. The last two rows “CF-SIM” refer to the stochastic car-following simulation results concerning the MACC-partial.

**Table 5:** Summary of results.^a^

**Experiments**	**KPI**	**Unit**	**ACC**	**MACC**	**Improvement**
CF-A	String stability	(−)	1.15	1.02	**11.30 %**
CF-A	Reaction time	(s)	1.20	1.20	**0.00 %**
CF-B	PFS*	(−)	0.98	0.93	**5.10 %**
CO	CFS*	(−)	0.44	0.14	**68.18 %**
CO	Minimum TTC	(s)	1.15	2.49	**116.52 %**
CO	Maximum deceleration	(m/s^2^)	−5.90	−2.36	**60.00 %**
CF-SIM	String stability	(−)	1.78	1.76 (20 %) 1.74 (50 %) 1.68 (100 %)	**1.04 % (20 %)** **2.52 % (50 %)** **5.60 % (100 %)**
CF-SIM	TET	(−)	0.0020	0.0016 (20 %) 0.0010 (50 %) 0.0003 (100 %)	**21.65 % (20 %)** **48.66 % (50 %)** **84.86 % (100 %)**

a There is no specific meaning linked to the use of bold

## 5 Discussion

Throughout the testing campaign, we were able to answer the research question: is multianticipation possible for vehicles that are widely available in the market, with the current state of technology? From the collected findings, we have sufficient evidence suggesting that at least one vehicle in the market is capable of detecting two leaders ahead provided that the right settings are enabled in the AEBS. The system proved to work remarkably stable when the second leader is moving. The same consideration did not apply to the static target in the cut-out cases.

The first results show that while multianticipation is possible, it seems to activate not for any change in the speed of the vehicle downstream, but when the separation between the two preceding vehicles is unsafely small. This is the first evidence that the commercial application is designed for safety and not for traffic flow. Moreover, the first evidence shows an increase in comfort, as smaller absolute values of deceleration and jerk are observed. Finally, the reaction to information gathered through multianticipation may be limited to a narrow range of decelerations. Deceleration values lower than 2 m/s^2^ were not observed during the multianticipation deceleration phase.

We have then discussed the potential impact on traffic flow and safety. Concerning the flow, we reported slightly better figures for the anticipative mode. Nonetheless, the system did not prove to be string stable regardless of the configuration. This finding further suggests that this specific multianticipation application has a stronger focus on safety than it has on traffic flow.

Considering, instead, safety, we compared the behavior in the anticipation mode with a safety performance model to quantitatively measure the increase in the safety level provided by the introduction of the anticipation mechanism by means of the PFS and CFS metrics. To carry out the safety performance assessment, we established, via simulation, a baseline model replicating the behavior that would have occurred without multianticipation according to the mentioned assumptions.

Overall, in the CF-B scenarios, multianticipation allowed reducing the PFS and, in particular, in two cases a PFS = 1 would have been reported in case multianticipation didn’t occur.

The COs analysis provided that the CFS was always 0 when multianticipation took place. Nonetheless, without multianticipation, three scenarios would have reported a criticality index higher than 0, implying the risk of deceleration harder than 2 m/s^2^ to avoid an accident, which is harder than some of the commercial ACC systems are designed to achieve.

The simulation investigation delivered interesting insights as it showed how even in ordinary driving scenarios, as of the “highD” dataset, even a partial multianticipation can prove helpful to increase safety. The impact on traffic flow turned out to be marginal as it could also be denoted from the experimental study thus qualitatively validating the modeling assumptions adopted. Additionally, the MACC resulted to be robust to different calibrations which can be important from a practical perspective as different OEMs might pursue different tuning strategies.

The study has thus remarkable implications for industry and academic practitioners. It proves in fact that multianticipation can be feasible as of today in contrast with connectivity which does not seem to be anywhere near large-scale market adoption. Although the currently experimented implementation is restricted to intervene during traffic conflicts, still it has managed to reduce the risk in a number of scenarios that could be easily faced during real-world driving. Policy-wise, the current legal frameworks do not typically force one specific technology to be featured on commercial vehicles. On the contrary, a technology-neutral approach is typically pursued where the policy-makers set the level of safety performance while letting the manufacturers free to select the best way to achieve such a threshold. The findings on assisted driving presented here have indeed the potential to inform current regulatory activities at the United Nations level concerning SAE J3016 L2 systems such as the Driver Control Assistance System (DCAS) UN-R171 (UNECE, 2024) and ADS systems such as the Automated Lane Keeping System (ALKS) UN-R157 (UNECE, 2023). Indeed both UN-Rs request a type of anticipative behavior. However, there is no specific operation characteristics requested in line with the technology neutrality principle. Instead, anticipative driving is considered to be fulfilled by checking the safety performance of the system using, for instance, a scenario-based approach. This makes such investigations important, as a robust benchmark for this type of anticipation is currently missing. The indication that risk can be reduced using multianticipation is very informative for stakeholders trying to characterize the preventability of rear-end collisions.

Nonetheless, the study is still affected by a number of limitations. The first one is that only one vehicle has been proven to feature multianticipation. Second, the methodological approach has been focused into characterizing the VUT specific implementation without a broader transportation-level implication study. We plan to address the first shortcoming by introducing testing practices to detect potential multianticipation capabilities being implemented in all the vehicles we regularly test. Concerning the second, we devoted to two separate publications (Donà et al., 2022, Donà et al., 2023) a simulation-based investigation where a candidate multianticipative ACC is tested against traditional ACC and CACC in heterogenous scenarios.

## 6 Conclusion

This paper has investigated the implementation of the multianticipation feature in the Tesla Model 3 MY2021 by means of car-following and cut-out experimental tests. In particular, a variant of the car-following test where the vehicle in the middle does not react to its leader has been conducted to stress the taking place of the multianticipation reaction.

We found that, in order to have any discernable multiple leader anticipation, the VUT’s AEBS was to be set into “Anticipative” mode. That allowed obtaining clear evidence that multianticipation occurred in both the variant car-following tests and cut-outs. In the normal operating mode, we could not find any indication suggesting multianticipation occurred.

When the suitable mode was enabled, we obtained consistent two leaders’ anticipation for the variant car-following tests and a slight improvement of the string stability margin for the traffic flow-oriented car-following experiments, despite the overall behavior did not prove to be string stable. Conversely, in the cut-out experimentations, the system did not prove to work as consistently and we could report multianticipation in less than half of the experiments. Possibly because of the static nature of the target which negatively affected the detection of the obstacle with respect to the car-following tests where the second leader was moving.

Overall, multianticipation proved to be an effective approach to address challenging driving scenarios more safely and, to some extent, improve the traffic flow with no need for inter-vehicle communication, such as in the CACC.

## Funding

The study was funded by the Joint Research Centre for the European Commission.

## CRediT authorship contribution statement

**Riccardo Donà:** Writing – original draft, Visualization, Methodology, Data curation, Conceptualization. **Konstantinos Mattas:** Writing – original draft, Visualization, Methodology, Data curation, Conceptualization. **Sandor Vass:** Writing – review & editing, Conceptualization. **Biagio Ciuffo:** Supervision, Funding acquisition, Conceptualization.

## Declaration of competing interest

The authors declare that they have no known competing financial interests or personal relationships that could have appeared to influence the work reported in this paper.

## Data Availability

Data will be made available on request.
